# Charting the regulatory landscape of the mouse genome *in vivo*

**DOI:** 10.1186/1756-8935-6-S1-O41

**Published:** 2013-03-18

**Authors:** Orsolva Symmons, Laurence Ettwiller, Sandra Ruf, Veli Uslu, Francois Spitz

**Affiliations:** 1 Developmental Biology Unit, European Molecular Biology Laboratory, Heidelberg, Germany; 2 Centre for Organismal Studies, University of Heidelberg, Heidelberg, Germany

## Background

Over the past years it has become apparent that a substantial part of mammalian gene regulation is achieved through the concerted action of long-range elements. Technical advances have greatly improved our ability to identify such elements, but still little is known about how they regulate gene expression mechanistically. It is particularly unclear to what extent genomic context influences the activity of regulatory elements, and therefore how authentically *in vitro* or transgene assays represent the actual activity of such elements. To characterise the regulatory architecture of genomic loci *in vivo*, we have developed GROMIT (Genome Regulatory Organisation Mapping with Integrated Transposons) [1]. GROMIT relies on the controlled mobilisation of a Sleeping Beauty transposon to distribute a lacZ reporter gene, acting as a regulatory sensor, throughout the mouse genome. The sensor integrates into the genome, without disrupting endogenous gene expression, and the precise location of insertions can be mapped.

## Results

Using GROMIT we have generated more than 1000 insertions, and determined the regulatory activities associated with approximately 700, distributed along all autosomes. This data revealed that tissue-specific regulatory activity is widely present throughout the genome, and is frequently organised into regulatory landscapes, large intervals of tens to hundreds of kilobases, within which insertions share expression patterns. By correlating the identified landscapes with published landmarks of gene regulation, as well as HiC data, we found that landscapes largely overlap with previously identified topological domains. In addition, since enhancers can activate the GROMIT sensor freely within, but not outside these landscapes, this indicates that conformational constraints may influence their activity.

Complementing this approach, a detailed study of the *Sonic hedgehog* locus allowed us to zoom-in on a single large landscape, and dissect the factors that influence enhancer activity. We produced a series of duplications and inversions that modify the distance and order of regulatory elements, without changing their primary sequence, and identified multiple configurations that led to mis-expression of *Sonic hedgehog*. This showed that communication between genes and their enhancers is greatly influenced by the genomic context. We speculate that the genomic structural rearrangements induce changes in epigenetic marks and three-dimensional organisation of the region that are predictive of the observed expression changes.

## Conclusions

GROMIT provides an *in vivo* experimental framework to manipulate the regulatory organisation of the mouse genome, which together with biochemical studies will shed light on how gene expression is precisely regulated in space and time.
